# Clinical application of VATS combined with 3D-CTBA in anatomical basal segmentectomy

**DOI:** 10.3389/fonc.2023.1137620

**Published:** 2023-02-21

**Authors:** Lening Zhang, Tuhui Wang, Yonggang Feng, Yizhao Chen, Chong Feng, Dongliang Qin, Chunshan Han

**Affiliations:** ^1^ Department of Thoracic Surgery, China−Japan Union Hospital of Jilin University, Changchun, China; ^2^ Department of emergency, China−Japan Union Hospital of Jilin University, Changchun, China

**Keywords:** VATS, 3D-CTBA, thoracic surgery, basal segment resection, lung cancer

## Abstract

**Objective:**

This study aimed to summarize the clinical application experience of video-assisted thoracic surgery (VATS) combined with three-dimensional computed tomography-bronchography and angiography (3D-CTBA) in anatomical basal segmentectomy.

**Methods:**

Clinical data of 42 patients who underwent bilateral lower sub-basal segmentectomy by VATS combined with 3D-CTBA in our hospital from January 2020 to June 2022 were retrospectively analyzed; the patients included 20 males and 22 females, with a median age of 48 (30–65) years. Combined with the preoperative enhanced CT and 3D-CTBA techniques to identify the altered bronchi, arteries, and veins during the operation, the anatomical resection of each basal segment of both lower lungs was completed through the fissure approach or inferior pulmonary vein approach.

**Results:**

All operations were successfully completed without conversion to thoracotomy or lobectomy. The median operation time was 125 (90–176) min, the median intraoperative blood loss was 15 (10–50) mL, the median postoperative thoracic drainage time was 3 (2–17) days, and the median postoperative hospital stay was 5 (3–20) days. The median number of resected lymph nodes was 6 (5–8). There was no in-hospital death. Postoperative pulmonary infection occurred in 1 case, lower extremity deep vein thrombosis (DVT) in 3 cases, pulmonary embolism in 1 case, and persistent air leakage in the chest in 5 cases, all of which were improved by conservative treatment. Two cases of pleural effusion after discharge were improved after ultrasound guided drainage. Postoperative pathology showed 31 cases of minimally invasive adenocarcinoma, 6 cases of adenocarcinoma *in situ* (AIS), 3 cases of severe atypical adenomatous hyperplasia (AAH), and 2 cases of other benign nodules. All cases were lymph node-negative.

**Conclusion:**

VATS combined with 3D-CTBA is safe and feasible in anatomical basal segmentectomy; consequently, this approach should be promoted and applied in clinical work.

## 1 Introduction

More than 70% of cases of lung cancer are non-small cell lung cancer (NSCLC). Early NSCLC has slow proliferation of cancer cells and late tumor spread; however, there are no obvious symptoms in the early stage and this cancer is not easily detected. Consequently, most patients with NSCLC are in the middle and late stages when diagnosed, and the mortality rate is high (1). Currently, lung cancer is the malignant tumor with the highest incidence and mortality worldwide. The improvement of health awareness in individuals and the popularization of chest high-resolution computed tomography (HRCT) scanning for physical examination have facilitated the detection of many sub-centimeter pulmonary nodules characterized by ground glass opacity (GGO) (2). Video-assisted thoracic surgery (VATS) is the preferred treatment for early lung cancer. Compared with lobectomy, anatomical segmentectomy can reduce the scope of lung tissue resection on the basis of ensuring adequate resection margin, protect lung function and postoperative quality of life, and achieve a long-term prognosis that is not inferior to that of lobectomy (3). However, owing to the complex anatomical structure of the lung, it is difficult to identify the segmental arteries, veins, and bronchi near the segmental hilum, especially in the case of anatomical variation. Surgeons often rely on their experience to transection blood vessels and segmental bronchi; however, incorrect transection of the bronchus will lead to atelectasis, and incorrect transection of the vein will lead to inaccurate intersegmental plane, resulting in poor surgical effect (4). Three-dimensional computed tomography-bronchography and angiography (3D-CTBA) technology has the advantage of clearly displaying the anatomical structure of bronchi, pulmonary arteries, and veins through 3D images, which allows determination of congenital variation, helps doctors accurately locate lesions before surgery, increases the chance of accurate surgical resection, and reduces operation errors and tissue damage during surgery (5, 6). Among all pulmonary segmental resections, basal segmentectomy of bilateral lower lobes is the most challenging because there are many vessels and bronchi with frequent variations, which easily lead to unclear identification of segmental hilum and intra-segmental structures, and the adjacent relationship between segmental planes is more complex (7). The application of auxiliary 3D-CTBA technology in basal segment resection can greatly reduce false injury and increase the probability of correct and precise resection. This study summarizes some short-term results that have been achieved using this approach.

## 2 Materials and methods

### 2.1 Clinical data

A total of 42 patients were enrolled in the study, including 20 males and 22 females, with a median age of 48 (30–65) years. Preoperative chest HRCT was routinely performed to determine the size, nature, and location of the lesion, and to complete the surgical plan (including target segment to be resected, the resection range, and the variation of the target segment bronchus and vessel, etc.). The indications for basal segment resection were: 1) diameter of nodule ≤2 cm, solid component <50%, and high suspicion of early lung cancer; 2) consider benign tumor or oligometastatic tumor, not suitable for wedge resection; 3) no surgical contraindications before operation, with cardiopulmonary function, blood test, etc. all meeting the surgical indications; 4) informed consent obtained from the patients. Exclusion criteria: 1) intraoperative frozen pathology suggested invasive carcinoma or positive sampling of lymph node; 2) previous history of ipsilateral thoracic surgery; 3) distant organ metastasis.

### 2.2 3D-CTBA image processing

**Figure 1:**
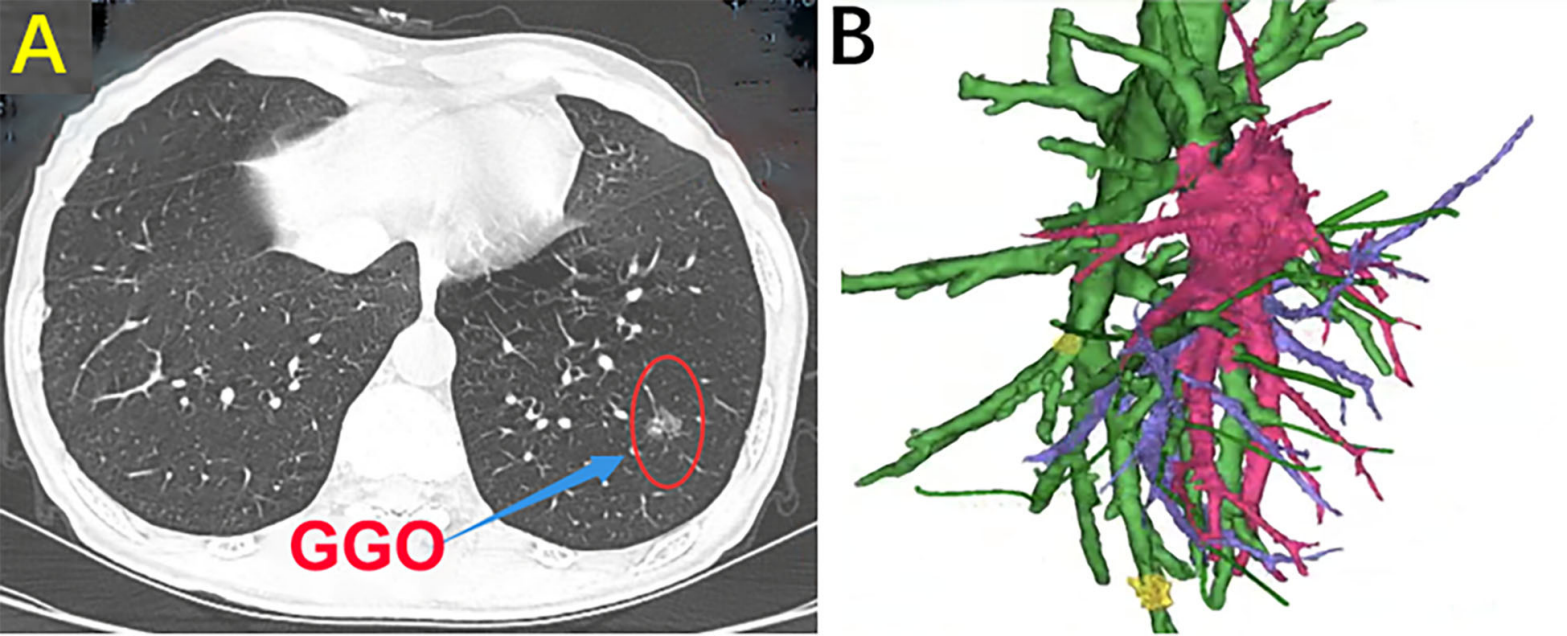
**(A)** Chest CT scan. **(B)** Three-dimensional computed tomography-bronchography and angiography (3D-CTBA).

Mimics 21.0 software was used to automatically calculate and generate the coronal and sagittal images after importing the original tomographic images. The transparency of each part was adjusted to determine the extent of resection after locating the position of the nodule in the target segment. Before the operation, the reconstructed images were transferred to a mobile computer. First, the presence of segmental vessel and segmental bronchus variation was determined. If there was variation, the precise location of the variation and whether it affected identification of the intersegmental plane was evaluated. In addition, whether the variation affected the margin was determined. During the operation, the 3D-CTBA images combined with a mobile computer can help surgeons complete the accurate segmentectomy (**Figure 1**).

### 2.3 Surgical methods

**Figure 2:**
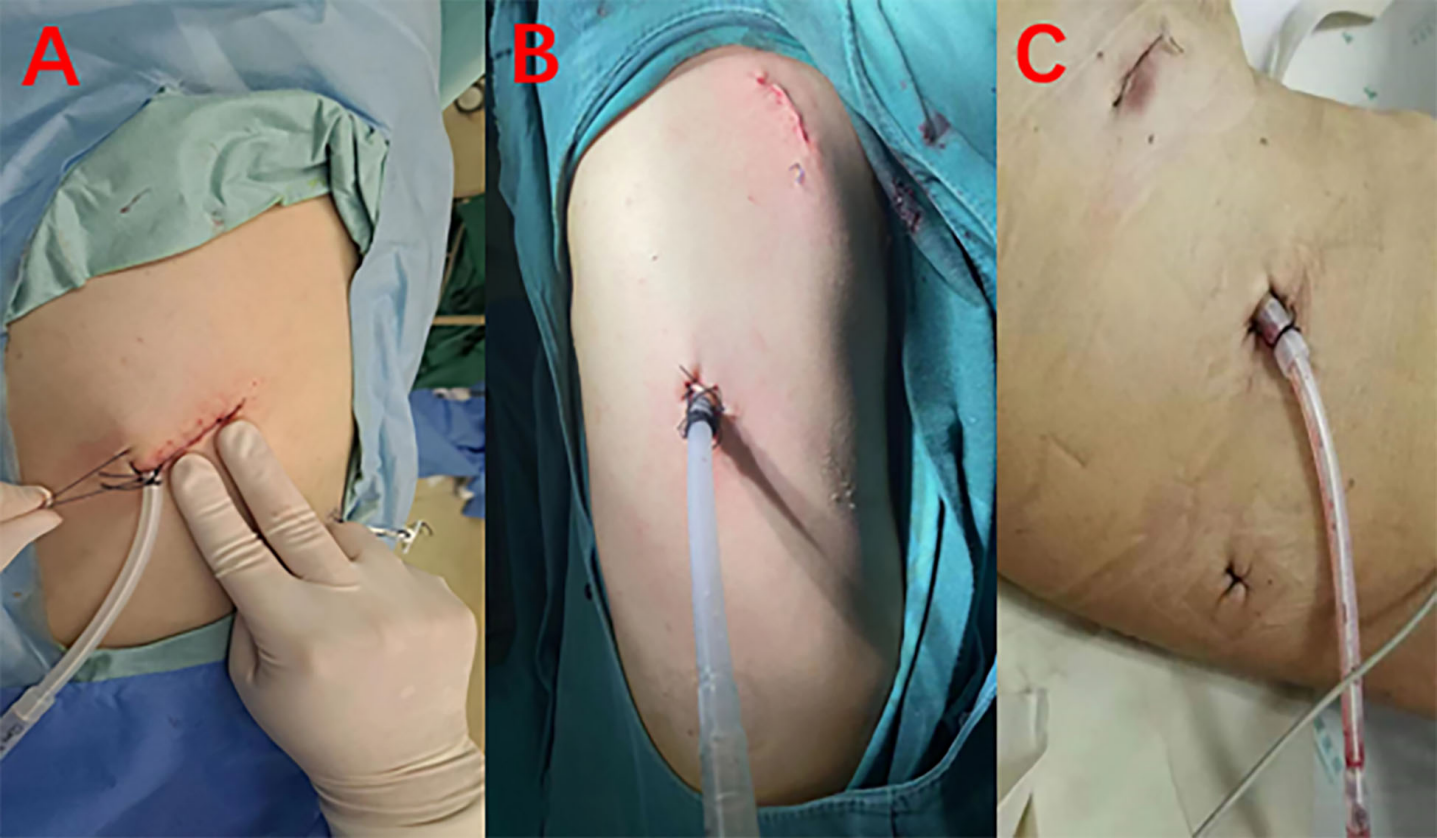
**(A)** Uniportal VATS. **(B)** Biportal VATS. **(C)** Triportal VATS.

After the patient was properly fixed, routine disinfection and drapting were performed. Incisions were made in the patient as follows: an incision of approximately 1 cm in the seventh intercostal space of the midaxillary line was used as the observation port; an incision of approximately 2–3 cm in the fourth or fifth intercostal space of the anterior axillary line (with or without an incision of approximately 1 cm in the ninth intercostal space of the posterior axillary line) was used as the operating port for multiportal VATS; an incision of approximately 4 cm in the fifth intercostal space of the anterior axillary line was made as the operating port for uniportal VATS (**Figure 2**). If there was adhesion, thoracolysis of pleural adhesion was initially performed, and then the situation of the lower pulmonary nodules was explored. After determining the general location of the nodules, the lower pulmonary ligament was routinely dissociated.

The choice of the oblique fissure approach or the inferior pulmonary vein approach depends on the development of the oblique fissure, and the order of treatment for the pulmonary artery, vein, and bronchus is also flexible according to the development of the fissure. In addition, the choice of the oblique fissure approach or inferior pulmonary vein approach corresponds to the anterior (anteromedial) basal segment or the lateral and posterior basal segment, respectively.

#### 2.3.1 Resection of the lateral and posterior basal segment

**Figure 3:**
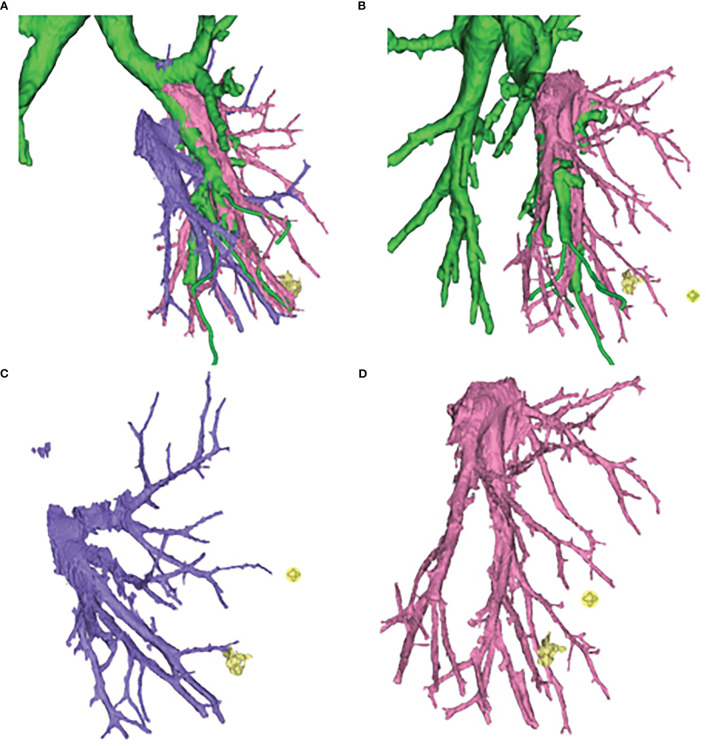
Three-dimensional computed tomography-bronchography and angiography (3D-CTBA) for GGO in S9. **(A)** Bronchi, artery and vein. **(B)** Bronchi and artery. **(C)** vein. **(D)** artery.

In general, even if the oblique fissure is well developed, the inferior pulmonary vein approach will be routinely performed in the lateral and posterior basal segment resection. Combined with preoperative 3D-CTBA image processing technology, the thoracic surgeons can identify the vessels and bronchi, and know whether there is a variation of vessels and bronchi (**Figure 3**).

**Figure 4:**
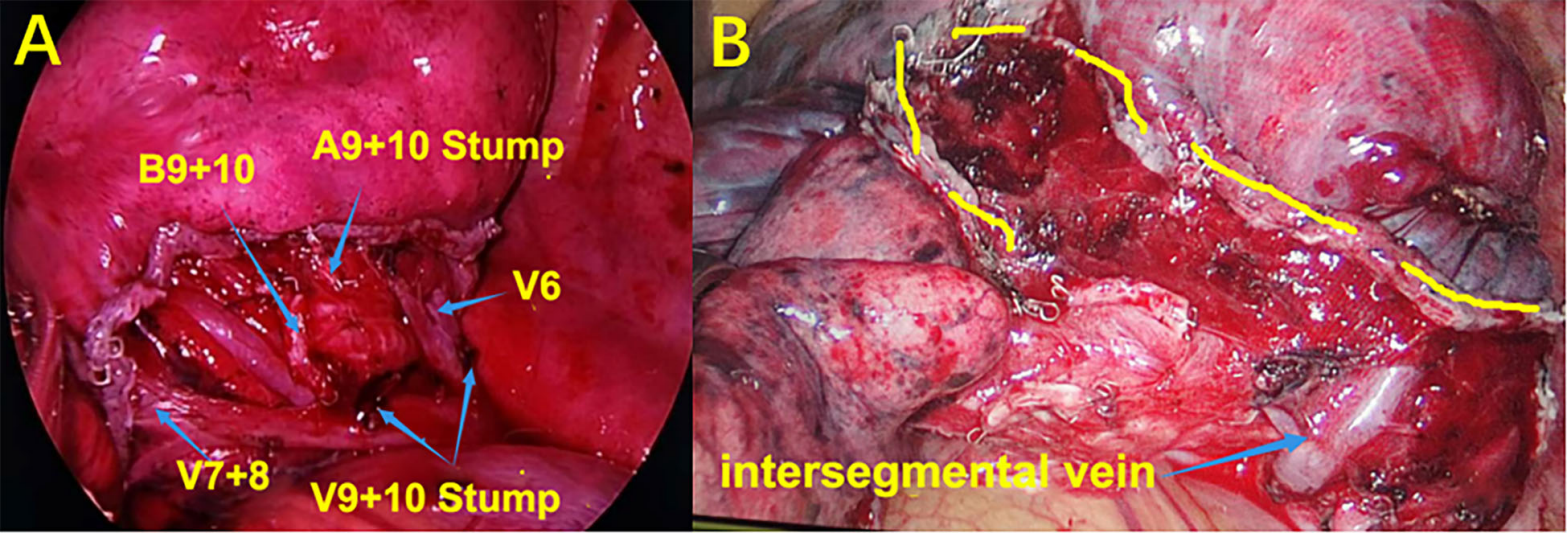
**(A)** S9+10 segmentectomy. **(B)** Intersegmental vein and intersegmental plane.

After confirming the dorsal branches of the inferior pulmonary vein (the dorsal veins often branch independently), the branches of the vein of the basal segment were fully dissociated, and then the location of the target segmental vein was identified. After ligation, the target segmental vein was removed by the ultrasonic scalpel. The target segmental bronchus was further explored and was transected with the endoscopic cutter stapler (if it was difficult to place the endoscopic cutter stapler, the target segmental bronchus could be ligated and the bronchial stump could be strengthened by Hem-O-Lok). The target segmental artery was further searched on the deep surface of the segmental bronchial stump. After ligation of No.7 surgical suture, the target segmental artery was removed with the ultrasonic scalpel. When it is difficult to identify the vessels and bronchi using the inferior pulmonary vein approach, the role of preoperative 3D-CTBA image processing technology will be significant, and the variation of vessels or bronchi can be located in advance to avoid accidental injury. Simultaneously, if the oblique fissure is well developed, the oblique fissure approach can be added to verify the vessels or bronchi that are difficult to identify. After separating the oblique fissure, the distribution of the segmental arteries and bronchi can be reconfirmed, so as to accurately remove the target segmental artery, vein, and bronchus, and accurately retain the intersegmental vein (the intersegmental vein is the natural boundary of the segments; separating the intersegmental lung tissue along the intersegmental vein can effectively reduce the air leakage and blood loss from lung tissue). Finally, at the end of these processes, accurate lateral and posterior basal segmentectomy is achieved (**Figure 4**).

#### 2.3.2 Resection of anterior basal segment or anteromedial basal segment

Routine dissection of the oblique fissure makes it easier to identify the arteries and bronchi. First, the oblique fissure was dissected, the target segmental artery was identified, and the basal segmental artery was removed with an endoscopic cutter stapler. Usually, arteries and bronchi distribute together, therefore the target segmental bronchus can be located by continuing exploration on the deep surface of the segmental arterial stump. After routine separation, the segmental bronchus was removed by an endoscopic cutter stapler. For dissection of the target segmental vein, both inferior pulmonary vein approach and oblique fissure approach can be used. Usually with the assistance of 3D-CTBA, the target segmental vein can be accurately resected, the intersegmental vein can be accurately preserved, and the anterior basal segment or anteromedial basal segment can be accurately resected.

## 3 Results

The operation was successfully completed in all patients, and there was no conversion to thoracotomy or lobectomy. All 42 patients underwent thoracoscopic basal segmentectomy, including 26 cases of simple basal segmentectomy and 16 cases of combined basal segmentectomy (the nodule located between the two basal segments). The median operation time was 125 (90–176) min, the median intraoperative blood loss was 15 (10–50) mL, the median postoperative thoracic drainage time was 3 (2–17) days, and the median postoperative hospital stay was 5 (3–20) days. The median number of resected lymph nodes was 6 (5–8). There was no in-hospital death. Postoperative pulmonary infection occurred in 1 case, lower extremity deep vein thrombosis (DVT) in 3 cases, pulmonary embolism in 1 case, and persistent air leakage in the chest in 5 cases, all of which were improved by conservative treatment. Two cases of pleural effusion after discharge were improved after ultrasound guided drainage. Postoperative pathology showed 31 cases of minimally invasive adenocarcinoma (MIA), 6 cases of adenocarcinoma *in situ* (AIS), 3 cases of severe atypical adenomatous hyperplasia (AAH), and 2 cases of other benign nodules. All cases were negative for lymph nodes. The specific surgical procedures and other clinical data are shown in **Table 1**.

**Table 1:** The specific surgical procedures and other clinical data.

Clinical parameter	Data
Age	
Gender	
Male	20
Female	22
Surgical options	
RS7	1
RS8	5
RS9	2
RS10	3
RS7+8	5
RS9+10	5
LS8	9
LS9	1
LS10	5
LS9+10	6
Operation time (min)	125 (90–176)
Lymph nodes removed	6 (5–8)
Intraoperative blood loss (mL)	15 (10–50)
Postoperative drainage time (days)	3 (2–17)
Postoperative hospital stay (days)	5(3–20)
Postoperative complications	
Pulmonary infection	1
Deep vein thrombosis	3
Pulmonary embolism	1
Persistent air leakage	5
Pleural effusion	2
Postoperative pathology	
MIA	31
AIS	6
AAH	3
Other benign tumors	2

## 4 Discussion

GGO is a common feature of early lung cancer encountered in the clinic. This kind of lung cancer often has low invasiveness and malignancy, slow growth, and good prognosis after surgical resection (8). GGO is represented by pure ground glass nodules and partial solid ground glass nodules, among which, partial solid nodules—especially those with a solid component <50%—are the standard indicators for segmentectomy. Anatomical segmentectomy has a long-term prognosis that is not inferior to that of lobectomy, and is better than lobectomy in terms of minimal trauma and protection of lung function (9).

Surgery is the first choice for the treatment of early lung cancer and can significantly prolong the survival rate of patients, with some patients being completely cured. The operation of lung cancer has developed from open surgery in the early days to minimally invasive thoracoscopic surgery used currently. At present, the commonly used minimally invasive thoracoscopic surgery for early GGO includes lobectomy, wedge resection, and segmentectomy. Pulmonary wedge resection is often used for the resection of benign lung tumors in clinical practice because it only targets superficial nodules, the scope of resection is limited, and it cannot be resected through the normal anatomical structure of the lung. For some elderly lung cancer patients with poor physical conditions, wedge resection can be the second-best choice. Lobectomy and lymph node dissection have played a long and unshakeable role in surgery for early lung cancer, and their effect and prognosis have been recognized for many years. However, this situation is changing as VATS is no longer a technical problem. The popularization of VATS in thoracic surgery and the advent of large data means there is no significant difference in the recurrence rate and prognosis survival rate between VATS and lobectomy for patients with early GGO (10). Under certain conditions, segmentectomy has the advantages of more precise resection, less trauma, more preservation of lung tissue, shorter recovery time, more preservation of lung function, and a significant decrease in the rate of infection. Okada et al. (11) found that for nodules less than 2 cm, there was no statistical difference in long-term survival rate between segmentectomy and lobectomy. Tsutani and colleagues (12) reported that for nodules less than 2 cm, there was no statistically significant difference in 3-year recurrence-free survival rate between segmentectomy and lobectomy. In addition, Zhao et al. (10) found that for nodules less than 2 cm, there was no statistically significant difference in 5-year recurrence-free survival rate between segmentectomy and lobectomy, and Altorki et al. (13) reported similar findings. From these studies, it can be concluded that the prognosis of thoracoscopic segmentectomy is not inferior to that of lobectomy. Therefore, for patients with early lung malignant tumors, anatomical segmentectomy has obvious effect and great significance (14). For the elderly and patients with normal basic cardiopulmonary function, thoracoscopic segmentectomy can retain more lung tissue in the anatomical scope and effectively protect lung function. Keenan et al. [30] retrospectively analyzed the pulmonary function of patients with stage I NSCLC and divided the surgical patients into two groups, who underwent lobectomy and segmentectomy, respectively. The mean preoperative forced expiratory volume in one second (FEV1) was 75.1% and 55.3% in the two groups, respectively, suggesting that the selected patients in the pulmonary segment group had relatively poor pulmonary function. One year after surgery, forced vital capacity (FVC) and FEV1 of patients in the lobar group decreased significantly, but there was no significant decline in the segmental group. Based on these two points, thoracoscopic segmentectomy has become a more popular surgical method by thoracic surgeons. In addition, the National Comprehensive Cancer Network, The National Health Commission of China (NCCN) guidelines also indicate that for most patients with early-stage NSCLC, anatomical segmentectomy is currently the main surgical method.

Previous scholars have questioned whether segmentectomy, compared with lobectomy, may increase the risk of tumor recurrence due to insufficient resection margin, resulting in a worse prognosis. Moreover, it has been reported that the recurrence rate after segmentectomy may be predominantly related to segmental location and margin width (15). Segmental surgery requires adequate safe resection margins. However, in thoracoscopic surgery, the lobe is in an atrophic state and it is difficult to identify the intersegmental veins and determine the safe resection margins. Simultaneously, compared with the lobar anatomy, the segmental anatomy of the lung is extremely complex, and there are many variations in the segmental arteries, veins, and bronchi, which are prone to accidental injury during surgery. Therefore, precise segmentectomy of the lung under a thoracoscope is difficult and risky. Moreover, there are many variations in the basal segment of the lung, and it is difficult to identify the segmental veins; consequently, accurate basal segmentectomy is the most difficult of all pulmonary segmental resections. Such variations cause great confusion for the identification of the basal segment veins. Exact identification of the veins will provide the best basis for the accurate segmentation of the intersegmental plane (16, 17); it is known that the intersegmental veins are the natural boundary marks between two lung segments and only after these veins are identified can the resection of the lung segment be accurate. In addition, for the resection of the lateral and posterior basal segments, the inferior pulmonary vein approach is the natural preferred approach; however, owing to variation in the bronchus and artery, such as the phenomenon of common trunk, the bronchus should not be blindly cut off during the operation, so as to avoid atelectasis or inaccurate resection. Proficiency in pulmonary segmental anatomy is crucial to the success of anatomical segmentectomy, especially the resection of basal lung segments with more challenging anatomy (18).

For a mature thoracic surgeon, it is necessary to understand the normal anatomical structure of the lung segments, but the morphological and spatial variation of the lung fissure, bronchus, and blood vessels that are not normally developed also need the help of CT imaging. Traditional two-dimensional CT images are not accurate for the anatomical structure of lung segments and subsegments, and it is also difficult to show the relationship between bronchi, blood vessels, and tumors without a 3D approach. Therefore, it cannot match the increasingly precise anatomical segmentectomy, which is significantly more challenging. In the past, thoracic surgeons needed to carefully dissect and repeatedly confirm the pulmonary vessels and bronchus during the operation to accurately understand the anatomical morphology of the vessels and bronchus, which not only increased the operation time and risk, but also increased the blindness and inaccuracy of pulmonary segmentectomy. Nowadays, in the auxiliary technology of 3D-CTBA, the bronchi, vessels, and nodules can be directly reconstructed in three dimensions before the operation, showing the volume of each lobe and segment, non-invasively showing the distance and 3D relationship between the primary tumor and the intersegmental veins, and safely reflecting the relationship between the resection margin and the pulmonary vessels. Therefore, the assistance of 3D-CTBA will help doctors identify anatomical variations before surgery, make surgical plans in advance, achieve accurate separation of vessels and bronchus during surgery, and accurately identify the intersegmental plane. Collectively, these benefits to the surgeons can effectively reduce the occurrence of postoperative air leakage and blood loss, shorten the operation time (19), reduce the accidental injury rate, and achieve better treatment effects. The application of 3D-CTBA can also ensure that the lung tissue is accurately cut according to the intersegmental plane, which naturally improves the probability of accurate resection, retains more normal lung tissue, preserves the lung function of the patient to the greatest extent, and reduces the risk of postoperative hypoxemia and respiratory failure. Therefore, thoracoscopic segmentectomy under 3D-CTBA is beneficial to the recovery of pulmonary function and effectively reduces the occurrence of postoperative complications. In this study, the 3D-CTBA simulated preoperative imaging was not 100% consistent with the actual anatomy during the operation. This discrepancy may be explained by a number of possibilities. Firstly, in some patients who are bed-bound for a long time, some of the bronchi on the back of body, such as the posterior segment of the right upper lobe, are chronically compressed and may not be able to be imaged on 3D images. Secondly, during thoracoscopic surgery, we generally take the lateral position and one-side lung ventilation, and lobes on the surgical side are in a collapsed state. Therefore, there will be some differences between the preoperative evaluation images of 3D-CTBA and the actual bronchi, arteries, and veins seen during the operation. There is also the possibility that vessels smaller than 2 mm in diameter are missing in the imaging of 3D-CTBA. In these cases, thoracic surgeons need to have clear theoretical knowledge and considerable experience.

As mentioned above, the accurate preservation of intersegmental veins is a natural and potential dividing line between subsegments of the lung. Splitting the lung tissue along the intersegmental veins greatly reduces the risk of postoperative air leakage, coupled with the application of biological glue and resistance to block the air leakage of lung tissue. In some cases, there will still be persistent air leakage after surgery, even after the above process (20). At this time, if the lung is well inflated, a 50% glucose solution (combined with lidocaine for pain relief) is used for pleural injection to promote pleural adhesion to treat air leakage after segmentectomy.

Most reports of thoracoscopic segmentectomy are usually limited to the lung segments with relatively simple anatomy, and there are few introductions to the more difficult segmental resections such as that of the basal segment. Basal segment resection is challenging because there are adjacent lung segments between each lung segment. When dividing the subbronchus, especially B9 or B10, because the angle of dividing the subsegmental bronchus is tricky, it is often necessary to add an auxiliary surgical incision to complete the operation. Moreover, if there is accidental bleeding when separating the blood vessels, it is also necessary to add an auxiliary surgical incision to complete the operation. When performing S9 surgery, it is necessary to accurately locate the intersegmental plane between the adjacent S8 and S10. If there is a common trunk or abnormal development of S9, it is easy to mis-cut or multi-cut the bronchus, arteries, and veins. In addition, it is necessary to ensure the safety range of the surgical margin (the distance between the nodule and the cutting edge should be >2 cm), and this can be confirmed by preoperative 3D-CTBA. Segmentectomy of S9+10 is often performed because this only involves the intersegmental plane between S8 and S9 and the intersegmental plane between S6 and S10 (21), which can effectively reduce the operation time. Therefore, if anatomical variation or margin safety problems are encountered during segmentectomy of S9, the surgeon will actively seek resection of the combined segment S9+10. S7 and S8 of the left pulmonary lower lobe naturally share the same trunk, combined with the natural boundary of oblique fissure, so S7+8 resection of the left pulmonary lower lobe is relatively easy in all basal segmental resections. Generally, the anterior oblique fissure is still well developed, so LS7+8 resection *via* oblique fissure approach is more common. Similarly, owing to the existence of the natural boundary of the oblique fissure, it is more convenient to take the oblique fissure approach in S8 of the right lung. Unlike B7 in the left lung, B7 in the right lung develops alone, “surrounded by mountains on three sides”, and S7 has a small volume. Therefore, cases of S7 resection alone are rare, and the combined subsegmental resection of RS7+8 is more common. The approach for the resection of both S9 and S10 is still the inferior pulmonary vein approach, which is more reliable (22). If the oblique fissure is still well developed, the oblique fissure approach can be added to verify the course of blood vessels and bronchi in cases where preoperative 3D-CTBA indicate variation, so as to increase the success rate of surgery.

This study does have some limitations and biases. Firstly, the sample size was small. Furthermore, the short follow-up time makes it impossible to evaluate the long-term efficacy of 3D-CTBA in thoracoscopic segmentectomy, and to analyze whether there is a difference in the long-term efficacy between the two groups. In addition, some preoperative CT was not enhanced CT, which resulted in unclear display of small vascular branches reconstructed by 3D-CTBA. The following aspects should be strengthened in future research. (1) Increase the number of patients, establish a 3D-CTBA data model of patients, analyze the anatomical morphology of patients, and provide data and image reference for other relevant medical departments. (2) This study is a retrospective study, and it is the preliminary results and experience obtained from a small sample size. In the future, we will expand the sample size, reduce the error, and conduct prospective studies to further verify the recurrence rate and long-term efficacy of 3D-CTBA in thoracoscopic segmentectomy, and analyze whether there is any difference between the two groups. (3) Comparative studies between single lung segments should also be performed to avoid errors caused by anatomical and surgical differences between different lung segments. (4) Further research on the application of 3D-CTBA in thoracic surgery, especially in the field of training young doctors is also needed.

In conclusion, VATS combined with 3D-CTBA for basal segment resection of the lower lung is clear and relatively simple to operate. 3D-CTBA can clarify the variation of vessels and bronchi before the operation, increase the accuracy of surgical resection, effectively preserve lung function, and reduce postoperative complications. In this study, the assistance of 3D-CTBA combined with the inferior pulmonary vein approach or/and oblique fissure approach resulted in successful completion of all operations, without conversion to thoracotomy and lobectomy, and all patients were discharged safely. This study proved that the technology is safe and feasible and can be widely applied in clinical work.

## Data availability statement

The original contributions presented in the study are included in the article/supplementary material. Further inquiries can be directed to the corresponding author.

## Ethics statement

The studies involving human participants were reviewed and approved by Ethics Committee of China−Japan Union Hospital of Jilin University. The patients/participants provided their written informed consent to participate in this study.

## Author contributions

CH put forward the theoretical concept. CH and LZ established the methodology. LZ, TW, YF, YC, CF, and DQ wrote the manuscript. CH, and LZ supervised the study. All authors contributed to the article and approved the submitted version.

## References

[1] Duma N Santana-Davila R Molina JR. Non-small cell lung cancer: Epidemiology, screening, diagnosis, and treatment. Mayo Clin Proc (2019) 94(8):1623–40. doi: 10.1016/j.mayocp.2019.01.013 31378236

[2] Hu W Zhang K Han X Zhao J Wang G Yuan S. Three-dimensional computed tomography angiography and bronchography combined with three-dimensional printing for thoracoscopic pulmonary segmentectomy in stage IA non-small cell lung cancer. J Thorac Dis (2021) 13(2):1187–95. doi: 10.21037/jtd-21-16 PMC7947531 33717591

[3] Nex G Schiavone M De Palma A Quercia R Brascia D De Iaco G. How to identify intersegmental planes in performing sublobar anatomical resections. J Thorac Dis (2020) 12(6):3369–75. doi: 10.21037/jtd.2020.01.09 PMC7330755 32642262

[4] Zhang J Zhu Y Li H Yu C Min W. Vats right posterior segmentectomy with anomalous bronchi and pulmonary vessels: A case report and literature review. J Cardiothorac Surg (2021) 16(1):1–5. doi: 10.1186/s13019-021-01420-2 33781306 PMC8008534

[5] Zhao X Zhao B Yao S Ding K. Clinical application of three-dimensional printing-assisted arthroscopic reconstruction of medial patellofemoral ligament to treat recurrent patellar dislocation in adolescents. Asian J Surg (2020) 43(12):1191–3. doi: 10.1016/j.asjsur.2020.09.005 32994113

[6] She X Gu Y Xu C Song X Li C Ding C. Combining 3D-CTBA and 3D-VATS single-Operation-Hole to Anatomical segmentectomy in the treatment of non-small cell lung cancer. Zhongguo fei ai za zhi = Chin J Lung Cancer (2017) 20(9):598–602. doi: 10.3779/j.issn.1009-3419.2017.09.02 PMC5973374 28935012

[7] Gao L Lin JH Yu SB Shen ZM Kang MQ. Application of 3D reconstruction technique in thoracoscopic anabolic resection of posterior basal segment of lung. Chin J Med Med (2019) 21(11):1605–8.

[8] Hirai Y Fujimori T Kasagawa T Ishii N Yasukawa T Kusashio K. Surgical resection of a solitary pulmonary nodule in a patient with breast cancer-a case report. Gan to kagaku ryoho. Cancer chemotherapy (2019) 46(13):2084–6. 32157067

[9] Herrmann D Gencheva-Bozhkova P Oggiano M Hecker E. Thoracoscopic sleeve segmentectomy for bipulmonal non-small-cell lung cancer with curative approach. Interact Cardiov Th. (2020) 31(5):737–9. doi: 10.1093/icvts/ivaa155 33057724

[10] Zhao R Shi Z Cheng S. Uniport video assisted thoracoscopic surgery (U-VATS) exhibits increased feasibility, non-inferior tolerance, and equal efficiency compared with multiport VATS and open thoracotomy in the elderly non-small cell lung cancer patients at early stage. Medicine (2019) 98(28). doi: 10.1097/MD.0000000000016137 PMC6641850 31305396

[11] Okada M Koike T Higashiyama M Yamato Y Kodama K Tsubota N. Radical sublobar resection for small-sized non-small cell lung cancer: A multicenter study. J Thorac Cardiovasc Surg (2006) 132(4):769–75. doi: 10.1016/j.jtcvs.2006.02.063 17000286

[12] Tsutani Y Miyata Y Nakayama H Okumura S Adachi S Yoshimura M. Oncologic outcomes of segmentectomy compared with lobectomy for clinical stage IA lung adenocarcinoma: Propensity score-matched analysis in a multicenter study. J Thorac Cardiovasc Surg (2013) 146(2):358–64. doi: 10.1016/j.jtcvs.2013.02.008 23477694

[13] Altorki NK Yip R Hanaoka T Baure T Aye R Kohman L. Sublobar resection is equivalent to lobectomy for clinical stage 1A lung cancer in solid nodules. J Thorac Cardiovasc Surg (2014) 147(2):754–62. doi: 10.1016/j.jtcvs.2013.09.065 24280722

[14] Echavarria MF Cheng AM Velez-Cubian FO Ng EP Moodie CC Garrett JR. Comparison of pulmonary function tests and perioperative outcomes after robotic-assisted pulmonary lobectomy vs segmentectomy. Am J Surg (2016) 212(6):1175–82. doi: 10.1016/j.amjsurg.2016.09.017 27823756

[15] Sienel W Stremmel C Kirschbaum A Hinterberger L Stoelben E Hasse J. Frequency of local recurrence following segmentectomy of stage IA non-small cell lung cancer is influenced by segment locatisation and width of resection margins - implications for patient selection for segmentectomy. Eur J Cardio-Thorac (2007) 31(3):522–7. doi: 10.1016/j.ejcts.2006.12.018 17229574

[16] Yang S Guo W Chen X Wu H Li H. Early outcomes of robotic versus uniportal video-assisted thoracic surgery for lung cancer: A propensity score-matched study. Eur J Cardio-Thorac (2018) 53(2):348–52. doi: 10.1093/ejcts/ezx310 28957995

[17] Xie BH Sui TY Qin Y Miao SC Jiao WJ. Short-term effects of video-assisted thoracoscopic segmentectomy on patients with early non-small cell lung cancer. Chin J Lung Cancer (2019) 22(12):767–71. 10.3779/j.issn.1009-3419.2019.12.06 PMC6935034 31874672

[18] Gossot D Seguin-Givelet A. Anatomical variations and pitfalls to know during thoracoscopic segmentectomies. J Thorac Dis (2018) 10:S1134–44. doi: 10.21037/jtd.2017.11.87 PMC5949391 29785286

[19] Wang M Lv H Wu T Gao W Tian Y Gai C. Application of three-dimensional computed tomography bronchography and angiography in thoracoscopic anatomical segmentectomy of the right upper lobe: A cohort study. Front Surg (2022) 9. doi: 10.3389/fsurg.2022.975552 PMC9530257 36204338

[20] Felip E Rosell R Antonio Maestre J Rodríguez-Paniagua JM Morán T Astudillo J. Preoperative chemotherapy plus surgery versus surgery plus adjuvant chemotherapy versus surgery alone in early-stage non-Small-Cell lung cancer. J Clin Oncol (2010) 28(19):3138–45. doi: 10.1200/JCO.2009.27.6204 20516435

[21] Shimizu K Mogi A Yajima T Nagashima T Ohtaki Y Obayashi K. Thoracoscopic subsuperior segment segmentectomy. Ann Thorac Surg (2017) 104(5):E407–10. doi: 10.1016/j.athoracsur.2017.07.007 29054241

[22] Pu Q Liu CW Guo CL Mei J Liu L. Stem-branch: A novel method for tracking the anatomy during thoracoscopic S9-10 segmentectomy. Ann Thorac Surg (2019) 108(5):E333–35. doi: 10.1016/j.athoracsur.2019.05.046 31288018

